# Chemical Compositions of the Volatile Oils and Antibacterial Screening of Solvent Extract from Downy Lavender

**DOI:** 10.3390/foods8040132

**Published:** 2019-04-19

**Authors:** Chang Ha Park, Ye Eun Park, Hyeon Ji Yeo, Se Won Chun, Thanislas Bastin Baskar, Soon Sung Lim, Sang Un Park

**Affiliations:** 1 Department of Crop Science, Chungnam National University, 99 Daehak-ro, Yuseong-gu, Daejeon 34134, Korea; parkch804@gmail.com (C.H.P.); yeney1996@cnu.ac.kr (Y.E.P.); guswl7627@gmail.com (H.J.Y.); seaw613@cnu.ac.kr (S.W.C.); bastinbt20@yahoo.com (T.B.B.); 2 Department of Food and Nutrition and Institute of Natural Medicine, Hallym University, Chuncheon 200-702, Korea; limss@hallym.ac.kr

**Keywords:** *Lavandula pubescens*, essential oil, antibacterial activity

## Abstract

The discovery of a new species exhibiting more effective antibacterial properties is necessary because of the demand on *Lavandula* species, which continues to increase in a variety of industries. *Lavandula pubescens* might be a good alternative, as it exhibits strong antibacterial activity. In this study, the chemical composition of the essential oils from different organs (flowers, leaves, stems, and roots) of *L. pubescens* was identified using gas chromatography-mass spectrometry. Furthermore, the antimicrobial activities of different solvent extracts (methanol, ethanol, diethyl ether, hexane, and ethyl acetate) and different organ (flower, leaf, stem, and root) extracts of *L. pubescens* were evaluated. Only the ethyl acetate extracts of *L. pubescens* exhibited antibacterial activity against all bacterial strains tested, including *Staphylococcus haemolyticus*, *Escherichia coli* (KF 918342), *Aeromonas hydrophila* (KCTC 12487), *E. coli* (ATCC 35150), *Cronobacter sakazakii* (ATCC 29544), and *Aeromonas salmonicida* (KACC 15136). In particular, the extracts exhibited significant activity against *S. haemolyticus.* Ethyl acetate extract of the leaf exhibited the best activity against all bacterial strains. This study provides valuable information on the chemical compositions in essential oils and antimicrobial properties of *L. pubescens*.

## 1. Introduction

*Lavandula pubescens*, also known as downy lavender, is an aromatic flowering plant belonging to the Lamiaceae family. *Lavandula*, consisting of approximately 39 known species, has been largely cultivated as ornamental plants for gardens and scenery use. This perennial plant is widely distributed in the Mediterranean, North Africa, Southwest Asia, western Iran, and eastern India [1]. The widely used and cultivated lavender species are *Lavandula angustifolia*, *Lavandula officinalis*, *Lavandula latifolia*, and *Lavandula vera. Lavandula pubescens* is a newly discovered species of lavender, which occurs in the Mediterranean. In recent years, the plants have been extensively studied as resources for medicine and aromatic products and used largely for their medicinal potentials, because these plants contain a number of bioactive compounds that act against human and plant pathogens [2], as well as compounds with various activities and properties [3].

Presently, *Lavandula* is widely studied because of its commercial use in the fragrance industry [4,5]. The plants have also been used as antibacterial, sedative, and antiviral agents in the pharmaceutical industries [6]. The essential oil of *Lavandula* species is presently used in food manufacturing industries for flavoring. *Lavandula* essential oil contains monoterpenes and is used in soaps, shampoos, mouthwashes, and household cleaners [7]. Previous studies reported that plant essential oils have natural preservative properties against food-borne microorganisms causing many infectious diseases in humans and widely contaminating meat and meat products [8] as well as fish-borne zoonotic parasites causing human anisakidosis [9,10]. Furthermore, these essential oils can improve prolongation of the shelf-life of perishable food products. Thus, *Lavandula* essential oil has been used in the food industry [11,12].

Essential oils, also called volatile or ethereal oils, are defined as an aromatic hydrophobic liquid, including a broad diversity of volatile compounds, and are derived from plant materials, such as the roots, stem, flowers, leaves, buds, seeds, and fruits [13]. Lavender essential oils have been used in complementary medicines, in cosmetics, or as food additives for centuries, because these oils have been traditionally regarded as antibacterial, antifungal, carminative, sedative, anti-inflammatory, and anti-depressive agents [14]. The chemical compounds in the oils, conferring such a biological property, can be classified into two main fractions: A volatile fraction that accounts for 90–95% of the whole oil and contains hydrocarbons (terpenes, sesquiterpenes, and diterpenes) and oxygenated derivatives generated from hydrocarbons containing phenols, aldehydes, esters, alcohol, oxides, and alcohols. The second non-volatile fraction forms 5–10% of the whole oil and includes waxes, hydrocarbons, sterols, fatty acids, carotenoids, psoralens, coumarins, and flavonoids [15,16]. The chemical composition varies greatly depending largely on species or extraction methods [17].

The antimicrobial activity of *Lavandula* has been widely studied [18,19,20]. Lavender plants have been used as a relaxant in aromatherapy [21,22]. Antioxidant and antiviral activities of *Lavandula* essential oil have been reported [23,24,25]. Therefore, this study aims to provide information on volatile constituents of in the essential oils from different organs (flowers, leaves, stems, and roots) of *L. pubescens* using gas chromatography-mass spectrometry (GC-MS) and the antibacterial activities of different plant parts of *L. pubescens*.

## 2. Materials and Methods

### 2.1. Chemicals

Ethanol (EtOH), methanol (MeOH), hexane, and ethyl acetate (EA) were purchased from Samchun Pure Chemical, Pyeongtaek, Korea. Diethyl ether (DE) and dimethyl sulfoxide (DMSO) were purchased from Sigma-Aldrich, Yongin, Korea. Luria-Bertani (LB) broth and streptomycin sulfate was purchased from MB cell, Seoul, Korea.

### 2.2. Plant Material and Extraction

**Figure 1:**
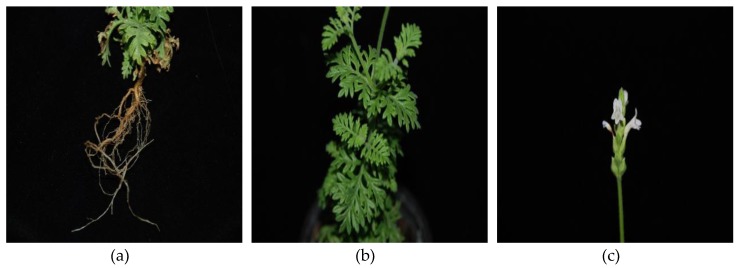
Photographs of the root (**a**), leaf and stem (**b**), and flower (**c**) of *Lavandula pubescens*.

The root, stem, leaf, and flower of *L. pubescens* were collected from the green house of Chungnam National University (Figure 1). The plants were washed under running tap water and dried under shade for three weeks. The extraction was performed according to the previous study [26]. The dried plants were then powdered using mortar and pestle and extracted with different solvents, including ethanol, methanol, hexane, ethyl acetate, and diethyl ether, respectively. The powdered sample (10 g) was soaked in 50 mL of the different solvents and incubated in an ultrasound bath (JAC-4020, KODO, Technical Research Co., Ltd., Hwaseong, Korea) for 1 day. The filtrate was then evaporated using a rotary vacuum evaporator (Laborota 4000, Heidolph Instruments Inc., Schwabach, Germany) and stored at 4 °C until needed for the antibacterial analysis. For screening of the different plant organs, the roots, stems, leaves, and flowers were collected, powdered, and dissolved in ethyl acetate for 1 day, after which each extract was collected and evaporated, as described above, and dissolved in the same solvent for further use.

### 2.3. Bacterial Strains and Cultivation

Six bacterial strains were used in this study: *Escherichia coli* (KF 918342), *Staphylococcus haemolyticus* (KCTC 3341), *Aeromonas hydrophila* (KCTC 12487), *Escherichia coli* (ATCC 35150), *Cronobacter sakazakii* (ATCC 29544), and *Aeromonas salmonicida* (KACC 15136). These strains were collected from the medicine department of Chungnam National University. The bacterial strains (*Escherichia coli* (KF 918342), *Staphylococcus haemolyticus* (KCTC 3341), *Escherichia coli* (ATCC 35150), *Cronobacter sakazakii* (ATCC 29544), and *Aeromonas salmonicida* (KACC 15136)) were cultured at 37 °C and the strain (*Aeromonas hydrophila* (KCTC 12487)) was cultured at 30 °C for 18–24 h in a 50 mL sample of LB broth prepared in a 250 mL conical flask medium placed on an orbital shaker. Optical density (OD) measurement was performed at 600 nm using a UV-1800 spectrophotometer (Shimadzu Corp., Kyoto, Japan). The culture flask was inoculated at 0.1 OD_600_ (optical density at 600 nm) with freshly prepared LB medium under the same culture conditions. The mid log phase bacterial cultures were used for the antibacterial studies. *Staphylococcus haemolyticus* (KCTC 3341) was the only gram-positive bacteria used in this study; all others were gram-negative bacteria.

### 2.4. GC-MS Analysis of L. pubescens

Volatile compounds were extracted and analyzed using a previously reported GC-MS method [27]. GC-MS analysis was performed on a 7820A GC/5977E MSD (Agilent, Santa Clara, CA, USA) with an HP-5 (30 m × 0.25 mm ID, film thickness 0.25 µm) fused-silica capillary column (Agilent, USA). Helium was used as the carrier gas at a flow rate of 1.0 mL/min. For GC-MS detection, an electron ionization system, with system energy of 70 eV, trap current of 250 µA, and an ion source temperature of 200 °C, was used. The oven temperature program was the same as that described for GC, and injections were used in the splitless mode. The column temperature was maintained at 35 °C for 2 min and programmed as follows: Increase from 50 to 250 °C at a rate of 10 °C/min and hold at 250 °C for 10 min. Fresh samples of each plant part (2.0 g) were placed in a 15 mL thermostatted vial that has a rubber septum. During the SPME extraction procedure, the SPME fiber was introduced for 12 h into the thermostatted vial (RT). For this analysis, a 1 cm, 50/30 µm polydimethylsiloxane/divinylbenzene/carboxen-coated fiber was used. The fiber was conditioned in a GC injection port for 1 min prior to use. The absorbed component was injected into a gas chromatograph by desorption at 250 °C for 2 min in the injector (splitless mode).

### 2.5. Antibacterial Screening

#### 2.5.1. Disk Diffusion Method

A 0.1 OD_600_ of the different overnight bacterial cultures was swabbed on a 25 mL LB agar plate. Whatman disk was then placed on the plates. A 30 µL sample of different solvent extracts of *L. pubescens* was added to the sterilized disk (Whatman No. 1 paper, 6 mm diameter) and incubated overnight at 37 °C. For screening of the different plant organs, the powdered samples were dissolved in ethyl acetate, and the resulting extract was added to the Whatman disk. Streptomycin (250 µg/mL) was used as the standard antibacterial agent. The experiment was performed in triplicate.

#### 2.5.2. Minimum Inhibitory Concentration

The minimum inhibitory concentration (MIC) of the *L. pubescens* extracts was established according to the method of Abdullah Al-Dhabi et al. [28] by using 96-well plates. A 1 mg/mL extract was dissolved in water with 2% DMSO. The initial extract concentration was 50 µL crude extract, which was then serially diluted two-fold (1.5625 to 50 µL). Each well had 100 µL of LB broth. Then, extracts were added at the concentrations described above. A 5 µL suspension containing 10^8^ CFU/mL of each of the six bacterial strains was added to the 96-well plate and incubated at 37 °C for 17 h. After the incubation time, the minimum inhibitor concentration was determined by the lowest visible growth in LB broth. The experiment was performed in triplicate.

## 3. Results

### 3.1. GC-MS Analysis of L. pubescens

Chemical composition of the essential oils of different organs of *L. pubescens*, such as the roots, stems, leaves, and flowers, were identified by GC-MS analysis. Most of the essential oils from different organs were characterized by the dominant presence of terpenes, including monoterpenes, diterpenes, and sesquiterpenes (Table 1). The oil from the leaves contained nine monoterpenes—neo-allo-ocimene (21.91%), *p*-cymenene (18.15%), terpinolene (3.31%), isothymol methyl ether (2.8%), *m*-cymene (2.1%), γ-terpinene (1.64%), carvacrol (0.78%), 2,5,5-trimethyl-1,3,6-heptatriene (0.76%), and *trans*-3-caren-2-ol (0.76%); nine sesquiterpenes—alloaromadendrene (2.45%), caryophyllene (2.45%), β-bisabolene (0.96%), α-muurolene (0.17%), (−)-β-elemene (0.16%), β-ylangene (0.13%), β-guaiene (0.11%), α-calacorene (0.11%), and acoradien (0.07%); and one diterpene—jolkinol D (0.16%), comprising 94.62% of the total leaf-derived essential oil.

The essential oil from the stems contained 10 monoterpenes—neo-allo-ocimene (15.68%), isothymol methyl ether (13.35%), γ-terpinene (11.09%), terpinolene (10.17%), *p*-cymenene (7.66%), *m*-cymene (2.89%), carvacrol (2.54%), *trans*-3-caren-2-ol (2.51%), 2,5,5-trimethyl-1,3,6-heptatriene (1.83%), and 4-terpinyl acetate (1.34%); and 10 sesquiterpenes—caryophyllene (9.97%), β-bisabolene (3.52%), β-ylangene (1.46%), longifolene (1.04%), α-cubebene (0.65%), β-eudesmene (0.58%), α-ylangene (0.38%), caryophyllene oxide (0.33%), aromandendrene (0.11%), and acoradien (0.08%), comprising 99.47% of the total stem-derived essential oil.

The essential oil from the roots contained 14 monoterpenes—*m*-cymene (49%), α-terpinene (4.22%), 4-terpinyl acetate (3.73%), (−)-4-terpineol (3.05%), *p*-xylene (2.46%), γ-terpinene (2.28%), d-limonene (2.0%), *p*-cymenene (1.99%), terpinolene (1.90%), α-terpinene (1.57%), carvacrol (0.41%) neo-allo-ocimene (0.36%), *trans*-3-caren-2-ol (0.33%), and isothymol methyl ether (0.35%); and 21 sesquiterpenes—(−)-calamenene (6.33%), α-gurjunene (3.30%), α-copaene (1.54%), β-eudesmene (1.21%), α-bergamotene (1.00%), caryophyllene (0.91%), δ-cadinene (0.88%), α-muurolene (0.86%), cyclosativene (0.81%), (−)-β-elemene (0.66%), β-copaene (0.61%), γ-cadinene (0.48%), α-calacorene (0.48%), α-cubebene (0.43%), β-guaiene (0.42%), α-ylangene (0.41%), acoradien (0.38%), caryophyllene oxide (0.19%), alloaromadendrene (0.16%), (+)-ledene (0.14%), and aromandendrene (0.07%), comprising 99.03% of the total root-derived essential oil.

The essential oil from flowers contained 13 monoterpenes—4-terpinyl acetate (16.99%), *p*-cymenene (11.66%), neo-allo-ocimene (8.55%), 1,3,5,5-tetramethyl-1,3-cyclohexadiene (2.95%), 2,5,5-trimethyl-1,3,6-heptatriene (2.75%), γ-terpinene (2.39%), *m*-cymene (1.31%), *trans*-3-caren-2-ol (1.12%), α-terpinene (0.87%), carvacrol (0.27%), *p*-xylene (0.15%), and d-limonene (0.08%); and 5 sesquiterpenes—longifolene (2.2%), β-bisabolene (0.72%), α-bergamotene (0.36%), caryophyllene (0.23%), and acoradien (0.07%), comprising 99.44% of the total flower-derived oil.

### 3.2. Antimicrobial Screening of L. pubescens

**Figure 2:**
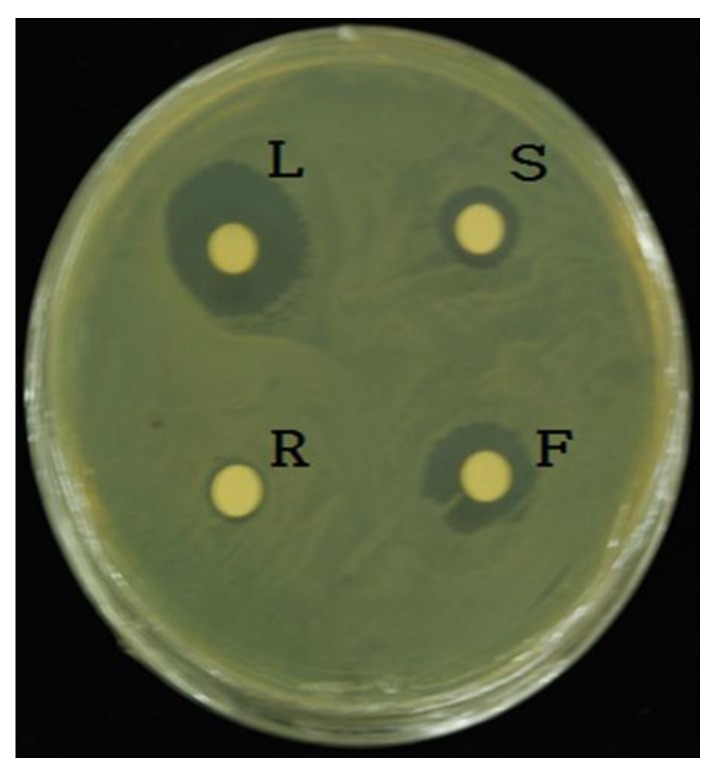
Representative image showing antibacterial activity against a bacterial pathogen. Leaf (L), flower (F), root (R), and stem (S) of *Lavandula pubescens*.

The antimicrobial screening of *L. pubescens* was performed using five different organic solvents. The ethyl acetate extract showed more antimicrobial activity against the six pathogenic bacteria because the disk diffusion method revealed that the ethyl acetate extract produced higher activity than that by the other organic solvents. In particular, *L*. *pubescens* possessed significant activity against *S. haemolyticus,* followed by *E. coli* (KF 918342), *A. hydrophila* (KCTC 12487), *E. coli* (ATCC 35150), *C. sakazakii* (ATCC 29544), and *A. salmonicida* (KACC 15136) (Table 2). Therefore, ethyl acetate was used for further studies on antimicrobial activities of *L. pubescens* because it had the strongest activity against all the bacteria. The antibacterial activity against bacterial pathogens using different plant organ extracts in agar plates is shown in Figure 2. The leaf extract exhibited the most powerful antibacterial activity against all bacterial strains, followed by the flower and stem extracts. On the other hand, the roots did not exhibit antimicrobial activity (Table 3).

### 3.3. Minimum Inhibitory Concentration of L. pubescens

The MIC of crude *Lavandula* extract was studied by the micro broth dilution method and the results are shown in Table 2. *Lavandula* ethyl acetate extract at different concentrations inhibited all the growth of all bacterial strain broths. The MIC for *E. coli* was 6.25 µL and for *S. haemolyticus*, *A. hydrophila,* and *A. salmonicida* was 12.5 µL. *Cronobacter sakazakii* inhibition was observed at 25 µL. Streptomycin showed better MIC values in comparison with those of the *Lavandula* ethyl acetate extracts. MIC values are shown in Table 4.

## 4. Discussion

In this study, 71 volatile compounds were identified in the essential oils derived from the roots, stems, leaves, and flowers of *L. pubescens* through GC-MS analysis. The most abundant constituents were terpenes, including monoterpenes, diterpenes, and sesquiterpenes. Similarly, previous studies have shown that terpene metabolites were the major components of essential oils from a variety of *Lavandula* species: Barocelli et al. reported a total of 23 constituents in *Lavandula hybrida* Reverchon “Grosso” essential oil, with major constituents such as linalool (33.4%), linalyl acetate (36.2%), camphor (7.6%), and 1,8-cineole (5.8%) [29]. Fenchone (55.79%), camphor (18.18%), 1,8-cineole (8.03%), and myrtenyl acetate (6.25%) were the major compounds among the 18 identified essential oil constituents of *Lavandula stoechas* L. (Spanish lavender) [30]. The major constituents in *L*. *angustifolia* essential oil were 1,8-cineole (44.9%), camphor (14.3%), β-phellandrene (5.0%), and α-pinene (4.7%) [31]. Shirugumbi et al. reported that *Lavandula bipinnata* essential oil had transcarveol (18.93%), pulegone (8.45%), camphor (7.09%), and menthol (5.89%) [32]. Spike lavender (*L. latifolia* Med.) and lavandin (*Lavandula × intermedia*) essential oils contained camphor (32.70%), 1,8-cineole (26.9%), and caryophyllene (4.88%) and 1,8-cineol (40.5%), linalool (33.1%), and camphor (8.17%), respectively [33]. Furthermore, Al-Badani et al. reported a total of 26 constituents in essential oil from the aerial parts of *L. pubescens*, with main components including carvacrol (72.7%), carvacrol methyl ether (7.0%), and caryophyllene oxide (5.9%) [34].

Ethyl acetate extract of *L. pubescens* tested in the present study showed strong antibacterial activity against the six bacterial strains tested. However, antibacterial activities of the extracts dissolved in the other solvents were not observed. This could be caused by the difference in the chemical composition of these extracts. Often, variations in chemical composition may result from differences in the extraction solvents, season, and presence of secondary metabolites [35]. Previous studies have shown that lavender plants contain a broad variety of terpenes, including monoterpenes, sesquiterpenes, and diterpenes, as well as phenolic compounds exhibiting antimicrobial activity [36,37,38].

Ethyl acetate extracts of *L. pubescens* possessed strong antibacterial activity against the six pathogenic bacteria, and *L. pubescens* leaves had the most powerful antimicrobial capacity. Our results are supported by those of previous studies showing that the essential oil from *Lavandula* species had antibacterial properties [39]. Hui et al. reported that *lavender* essential oil showed anti-bacterial activities against *E*. *coli* and *Staphylococcus aureus* [40]. Hossain reported that the essential oil of *L*. *angustifolia* was effective against all tested turtle-borne pathogenic bacteria: *A. hydrophila*, *Aeromonas caviae*, and *Aeromonas dhakensis* [41]. Furthermore, lavender essential oil nanoemulsions showed anti-bacterial activity against *C. sakazakii* [42]. The essential oil of *L*. *angustifolia* showed anti-bacterial activity against *S*. *aureus*, *E*. *coli*, *Citrobacter freundii*, *Enterobacter aerogenes*, *Propionibacterium acnes*, *Proteus vulgaris*, *Pseudomonas aeruginosa*, *Shigella sonnei*, and *Streptococcus pyogenes* [43]. Therefore, the essential oils from *Lavandula* plants are expected to possess important antibacterial properties against various bacterial species.

According to the World Health Organization (WHO), approximately 80% of the world’s population depends on herbal remedies for their primary healthcare [44,45]. This study showed the different chemical composition in essential oils of different parts of *L. pubescens* and the different antibacterial activity of ethyl acetate extracts of these plant parts. These properties could be successfully exploited to treat several diseases caused by bacterial infections. This may indicate that lavender species used in traditional remedies may possess beneficial biological activity. Thus, this study suggests the potential use of *L. pubescens* roots, stems, leaves, and flowers in traditional herbal medicine applications.

## 5. Conclusions

In this study, ethyl acetate was the most effective solvent for extracting a broad variety of compounds from *L. pubescens*, and its extract possessed strong antibacterial activity against *Escherichia coli* (KF 918342), *Staphylococcus haemolyticus* (KCTC 3341), *Aeromonas hydrophila* (KCTC 12487), *Escherichia coli* (ATCC 35150), *Cronobacter sakazakii* (ATCC 29544), and *Aeromonas salmonicida* (KACC 15136). In particular, the leaves exhibited the strongest antimicrobial activity against these bacteria among the different plant parts tested. Therefore, this study suggests that *L. pubescens* could be considered a good source for human health.

## Figures and Tables

**Table 1:** Compounds identified in the different organs of *Lavandula pubescens* using gas chromatography-mass spectrometry (GC-MS).

Volatile Compound	Peak Area (%)				
	RT ^1^	Leaf	Stem	Flower	Root
*p*-Xylene	5.22	ND ^2^	ND	0.15	2.46
d-Limonene	6.8	ND	ND	0.08	2
γ-Terpinene	7.37	1.64	11.09	2.39	2.28
α-Terpinene	7.49	ND	ND	0.33	4.22
1,3,5,5-Tetramethyl-1,3-cyclohexadiene	7.6	ND	ND	2.95	ND
*m*-Cymene	7.67	2.1	2.89	1.31	49
2,5,5-Trimethyl-1,3,6-heptatriene	7.81	0.76	1.83	2.75	ND
4-Terpinyl acetate	8.19	ND	1.34	16.99	3.73
Terpinolene	8.67	3.31	10.17	ND	1.9
*p*-Cymenene	8.74	18.15	7.66	11.66	1.99
*trans*-3-Caren-2-ol	9.11	0.76	2.51	1.12	0.33
(−)-4-Terpineol	10.13	ND	ND	ND	3.05
Neo-allo-ocimene	9.32	21.91	15.68	8.55	0.36
α-Terpinene	10.35	ND	ND	0.54	1.57
Isothymol methyl ether	11.06	2.8	13.35	ND	0.35
Carvacrol	11.97	0.78	2.54	0.27	0.41
α-Cubebene	12.58	ND	0.65	ND	0.43
α-Ylangene	12.9	ND	0.38	ND	0.41
α-Copaene	12.96	ND	ND	ND	1.54
(−)-β-Elemene	13.15	0.16	ND	ND	0.66
Alloaromadendrene	13.22	2.45	ND	ND	0.16
(+)-Ledene	13.41	ND	ND	ND	0.14
Longifolene	13.46	ND	1.04	2.2	ND
Caryophyllene	13.58	2.45	9.97	0.23	0.91
β-Ylangene	13.67	0.13	1.46	ND	ND
α-Bergamotene	13.7	ND	ND	0.36	1
Aromandendrene	13.82	ND	0.11	ND	0.07
Caryophyllene oxide	13.94	ND	0.33	ND	0.19
Acoradien	14.02	0.07	0.08	0.07	0.38
δ-Cadinene	14.23	ND	ND	ND	0.88
Cyclosativene	14.25	ND	ND	ND	0.81
α-Muurolene	14.3	0.17	ND	ND	0.86
β-Copaene	14.35	ND	ND	ND	0.61
β-Eudesmene	14.45	ND	0.58	ND	1.21
α-Gurjunene	14.53	ND	ND	ND	3.3
β-Bisabolene	14.6	0.96	3.52	0.72	ND
γ-Cadinene	14.74	ND	ND	ND	0.48
(−)-Calamenene	14.85	ND	ND	ND	6.33
β-Guaiene	15.03	0.11	ND	ND	0.42
α-Calacorene	15.11	0.11	ND	ND	0.48
Jolkinol D	20.22	0.16	ND	ND	ND
1,3,5,7-Cyclooctatetraene	5.57	0.85	ND	0.37	ND
(3E)-2,7-Dimethyl-3-octen-5-yen	6.05	ND	0.14	0.18	2.35
5,6-Dimethylene-1-cyclooctene	6.62	0.48	ND	0.02	ND
Benzaldehyde	6.79	1.22	ND	0.12	ND
1,2,3,4,5-Pentamethylcyclopentadiene	6.88	0.63	ND	0.85	ND
2,5-Dimethyl-3-methylene-5-heptadiene	7.15	ND	1.04	2.42	ND
α-Cumene hydroperoxide	7.71	1.45	2.05	0.13	ND
6-Methyl-5-(1-methylethylidene)-6,8-nonadien-2-one	7.72	8.25	2.66	6.47	ND
(3Z)-2,7-Dimethyl-3-octen-5-yne	8.04	0.55	ND	ND	ND
1,3-Dimethyl-1,5-cyclooctadiene	8.41	0.31	ND	19.76	ND
1-Methyl6-isopropylidene	8.6	6.18	2.51	14.94	ND
4-(1,5-Dihydro-2,4,3-benzodioxaborepin-3-yl) benzoic acid	9.04	0.36	ND	ND	ND
1-Amino-2-(4-chlorobenzoyl)-6,7,8,9-tetrahydro-5-methylthieno[2,3-c]isoquinoline	9.62	0.65	2.87	ND	0.61
Naphthalene	10.31	0.39	ND	ND	ND
6-Isopropenyl-3-(methoxymethoxy)-3-methyl-1-cyclohexene	10.34	0.62	ND	ND	ND
Benzothiazole	10.98	0.38	ND	1.42	ND
3,5,7-Trimethoxy-2-(4-methoxyphenyl)-4H-chromen-4-one	12.09	0.35	0.83	ND	0.18
δ-Valerolactone	14.06	0.44	ND	0.09	0.24
2,6-Di-*tert*-butylbenzoquinone	14.13	0.27	ND	ND	0.38
4,4,5,6-Tetramethyl-1,3-oxazinane-2-thione	16.23	3.06	ND	ND	ND
2-(3-Acetoxy-4,4,10,13,14-pentamethyl-2,3,4,5,6,7,10,11,12,13,14,15,16,17-tetradecahydro-1H-cyclopenta[a]phenanthren-17-yl)-propanoic acid	16.34	0.26	ND	ND	0.09
2,6-Di-*tert*-butylhydroquinone	16.74	0.65	ND	ND	ND
3,5-Bis(*tert*-butyl)-4-hydroxy-propiophenon	17.79	0.21	ND	ND	ND
(6-*tert*-Butyl-1,1-dimethyl-2,3-dihydro-1H-inden-4-yl)acetic acid	17.88	0.5	ND	ND	ND
2,4,6-Tri-*tert*-butylphenol	18.2	5.18	ND	ND	ND
1-(3,5-Di-*tert*-butyl-4-hydroxyphenyl)propan-1-one	18.95	0.52	ND	ND	ND
3,3,3′,3′,5,5,5′,5′-Octamethyl-1,1′-bi(1-cyclohexen-1yl)	19.23	0.29	0.2	ND	ND
3-Deoxyestradiol	19.49	0.14	ND	ND	0.15
1-Nitrosopyrrolidine	20.74	1.48	ND	ND	ND
3-Hydroxyspirost-8-en-11-one	28.36	ND	ND	ND	0.1
Total identified (%)		94.65	99.48	99.44	99.02

^1^ RT, Retention time. ^2^ ND, Not detected.

**Table 2:** Antibacterial activity of *Lavandula pubescens* using extracts with different solvents. Each value is the average of three trials ± standard deviation.

Strains	Zone of Inhibition(mm) from *L*. *pubescens* Extracts					
	MeOH ^1^	EtOH ^2^	DE ^3^	EA ^4^	Hexane	Streptomycin
*Escherichia coli* (KF 918342)	NA ^5^	NA	NA	21.3 ± 0.6	NA	27.6 ± 0.6
*Staphylococcus haemolyticus*	NA	NA	NA	24.0 ± 0.0	NA	26.3 ± 0.6
*Aeromonas hydrophila*	NA	NA	NA	21.0 ± 1.0	NA	27.0 ± 0.0
*Escherichia coli* (ATCC 35150)	NA	NA	NA	20.6 ± 1.1	NA	28.3 ± 0.6
*Cronobacter sakazakii*	NA	NA	NA	22.3 ± 0.6	NA	25.6 ± 0.6
*Aeromonas salmonicida*	NA	NA	NA	19.6 ± 1.5	NA	27.0 ± 1.0

^1^ MeOH, methanol. ^2^ EtOH, ethanol. ^3^ DE, di ethyl ether. ^4^ EA, ethyl acetate. ^5^ NA, no activity.

**Table 3:** Antibacterial activity of different organs of *Lavandula pubescens*. Each value is the average of three trials ± standard deviation.

Strains	Zone of Inhibition(mm) from *L*. *pubescens* Extracts			
	Flower	Leaf	Stem	Root
*Escherichia coli* (KF 918342)	17.6 ± 0.6	22.3 ± 0.6	15.3 ± 0.6	NA ^1^
*staphylococcus haemolyticus*	17.6 ± 0.6	23.0 ± 1.0	19.0 ± 1.0	NA
*Aeromonas hydrophila*	14.3 ± 1.5	16.0 ± 0.0	13.7 ± 0.6	NA
*Escherichia coli* (ATCC 35150)	15.3 ± 0.6	18.0 ± 1.0	13.0 ± 0.0	NA
*Cronobacter sakazakii*	13.7 ± 0.6	21.3 ± 1.1	13.6 ± 0.6	NA
*Aeromonas salmonicida*	14.7 ± 0.6	20.3 ± 0.6	12.3 ± 0.6	NA

^1^ NA, no activity.

**Table 4:** Antibacterial activity of different organs of *Lavandula pubescens*.

Strains	Minimum Inhibitory Concentration (MIC)	
	Compound (µL)	Standard (µg)
*Escherichia coli* (KF 918342)	6.25	25
*Staphylococcus haemolyticus*	12.5	50
*Aeromonas hydrophila*	12.5	50
*Escherichia coli* (ATCC 35150)	6.25	25
*Cronobacter sakazakii*	25	100
*Aeromonas salmonicida*	12.5	50
